# Supplementary material for: Top-down and bottom-up interactions rely on nested brain oscillations to shape rhythmic visual attention sampling
Source: PLoS Biol. 2025 Apr 10;23(4):e3002688. doi: 10.1371/journal.pbio.3002688 (PMC12037075; doi:10.1371/journal.pbio.3002688)
Supplement: S1 Fig — (A) Left panel: Raw differences in time-frequency plots of the posterior electrodes (O2, O1, POz, Oz, PO8, PO7, PO4, PO3) between grating-present and grating-absent condition (GRATING±). Frequency range for the analysis (y-axis) is from 5–80 Hz. Time range for the analysis (x-axis) is from −200–5,000 ms, where 0 is the time point of the grating onset. Right panel: Z-scores of the permutation-based analysis between grating-present and grating-absent condition. Significant clusters are framed with the black line. (B) Topographies of the significant clusters of the amplitude differences in the gamma frequency band (lower). diff = difference; dB = decibel; Hz = hertz; t = time. (DOCX) [file pbio.3002688.s001.docx]

| 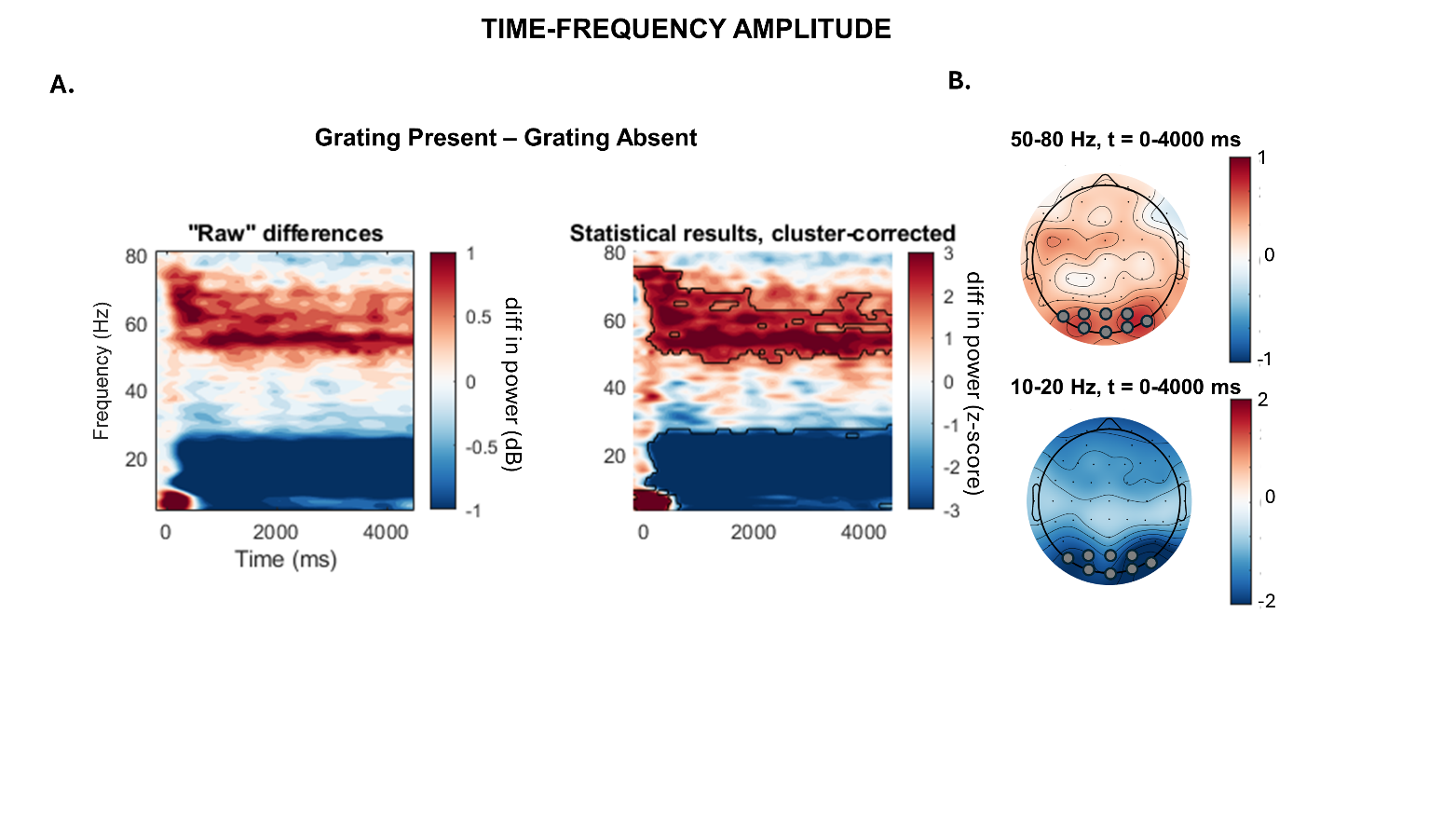 |
| --- |

**S1 Fig.** **Time-Frequency Analysis for the whole grating duration: Grating effects**. **A.** Left panel: Raw differences in time-frequency plots of the posterior electrodes (O2, O1, POz, Oz, PO8, PO7, PO4, PO3) between grating present and grating absent condition (GRATING+/-). Frequency range for the analysis (y-axis) is from 5-80Hz. Time range for the analysis (x-axis) is from -200 to 5000 ms, where 0 is the time point of the grating onset. Right panel: Z-scores of the permutation-based analysis between grating present and grating absent condition. Significant clusters are framed with the black line. **B.** Topographies of the significant clusters of the amplitude differences in the gamma frequency band (lower). diff = difference; dB = decibel; Hz=hertz; t=time.
