# Supplementary material for: Top-down and bottom-up interactions rely on nested brain oscillations to shape rhythmic visual attention sampling
Source: PLoS Biol. 2025 Apr 10;23(4):e3002688. doi: 10.1371/journal.pbio.3002688 (PMC12037075; doi:10.1371/journal.pbio.3002688)
Supplement: S2 Fig — Potential differences in the preferred phase lag angle differences between the electrode sites are not responsible for the obtained connectivity differences. Specifically, we did not find significant differences between the electrodes and frequencies where we found significant connectivity changes via wPLI in the grating-present (all ts < 1.702; all ps > 0.090) and grating-absent condition (all ts < 1.287; all ps > 0.203). (A) Grating-absent condition. Phase-angle differences for the FEF-TMS (left) and M1-TMS condition (right) in polar coordinates between one of the significant electrode pairs (AF8-PO4) at 16 Hz. Each line represents phase-angle differences of a single participant. (B) Grating-present condition. Phase-angle differences for the FEF-TMS (left) and M1-TMS condition (right) in polar coordinates between one of the significant electrode pairs (AF8-PO4) at 16 Hz. Each line represents phase-angle differences of a single participant. (DOCX) [file pbio.3002688.s002.docx]

| 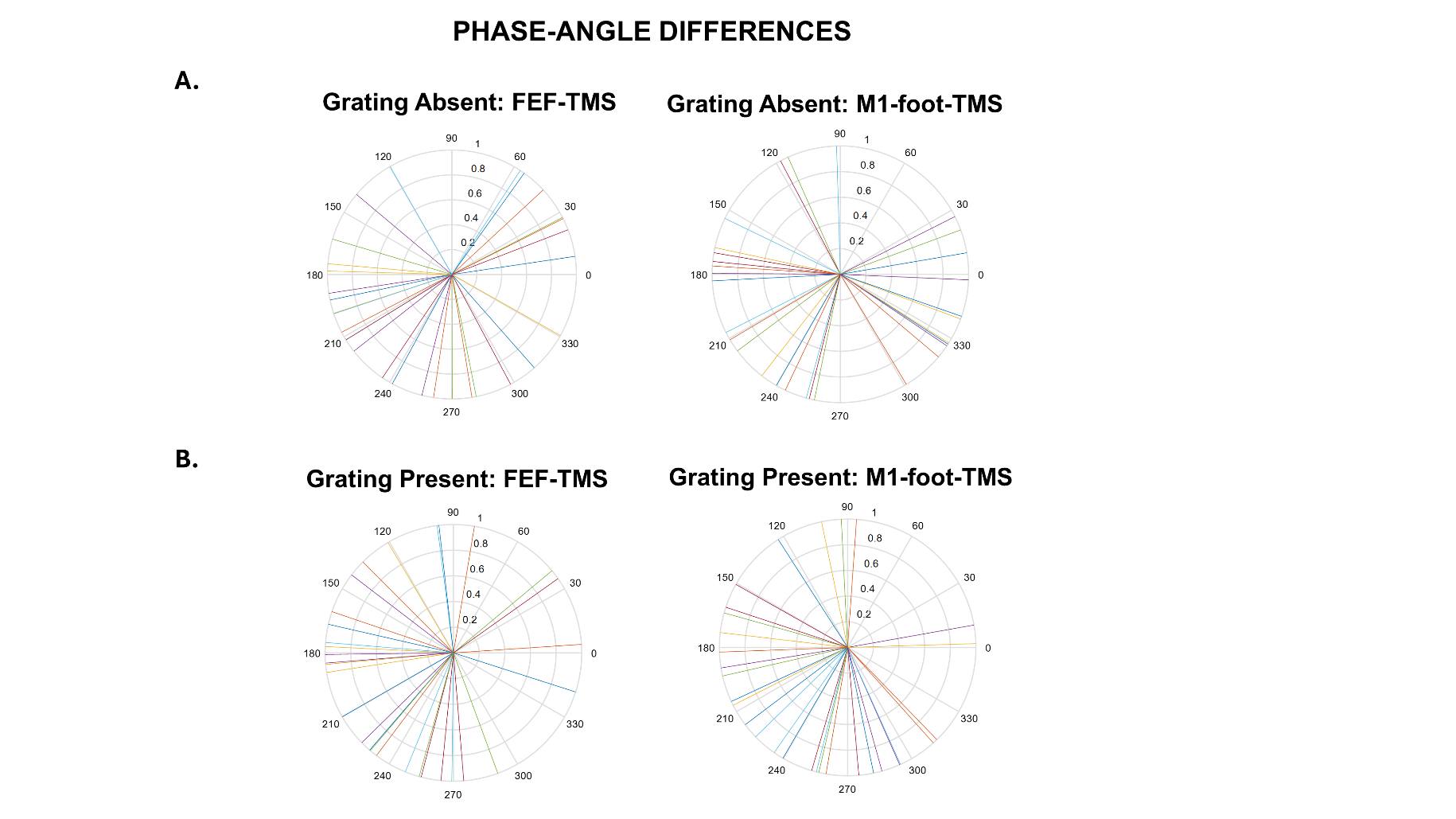 |
| --- |

**S2 Fig.** **Phase-Angle differences**. Potential differences in the preferred phase lag angle differences between the electrode sites are not responsible for the obtained connectivity differences. Specifically, we did not find significant differences between the electrodes and frequencies where we found significant connectivity changes via wPLI in the grating present (all ts < 1.702; all ps > .090) and absent condition (all ts < 1.287; all ps > .203). **A.** Grating absent condition. Phase angle differences for the FEF-TMS (left) and M1-TMS condition (right) in polar coordinates between one of the significant electrode pairs (AF8-PO4) at 16 Hz. Each line represents phase angle differences of a single participant. **B.** Grating present condition. Phase angle differences for the FEF-TMS (left) and M1-TMS condition (right) in polar coordinates between one of the significant electrode pairs (AF8-PO4) at 16 Hz. Each line represents phase angle differences of a single participant.
