# Supplementary material for: Top-down and bottom-up interactions rely on nested brain oscillations to shape rhythmic visual attention sampling
Source: PLoS Biol. 2025 Apr 10;23(4):e3002688. doi: 10.1371/journal.pbio.3002688 (PMC12037075; doi:10.1371/journal.pbio.3002688)
Supplement: S3 Fig — showing the presence of high alpha-low beta band activity which is a prerequisite for phase-analyses in these frequency bands. (A) Grating-absent condition. Power-frequency spectrum for the FEF-TMS (left) and M1-TMS condition (right) averaged across participants. Gray square represents the frequency window of interest. (B) Grating-present condition. Power-frequency spectrum for the FEF-TMS (left) and M1-TMS condition (right) averaged across participants. Gray square represents the frequency window of interest. (DOCX) [file pbio.3002688.s003.docx]

| 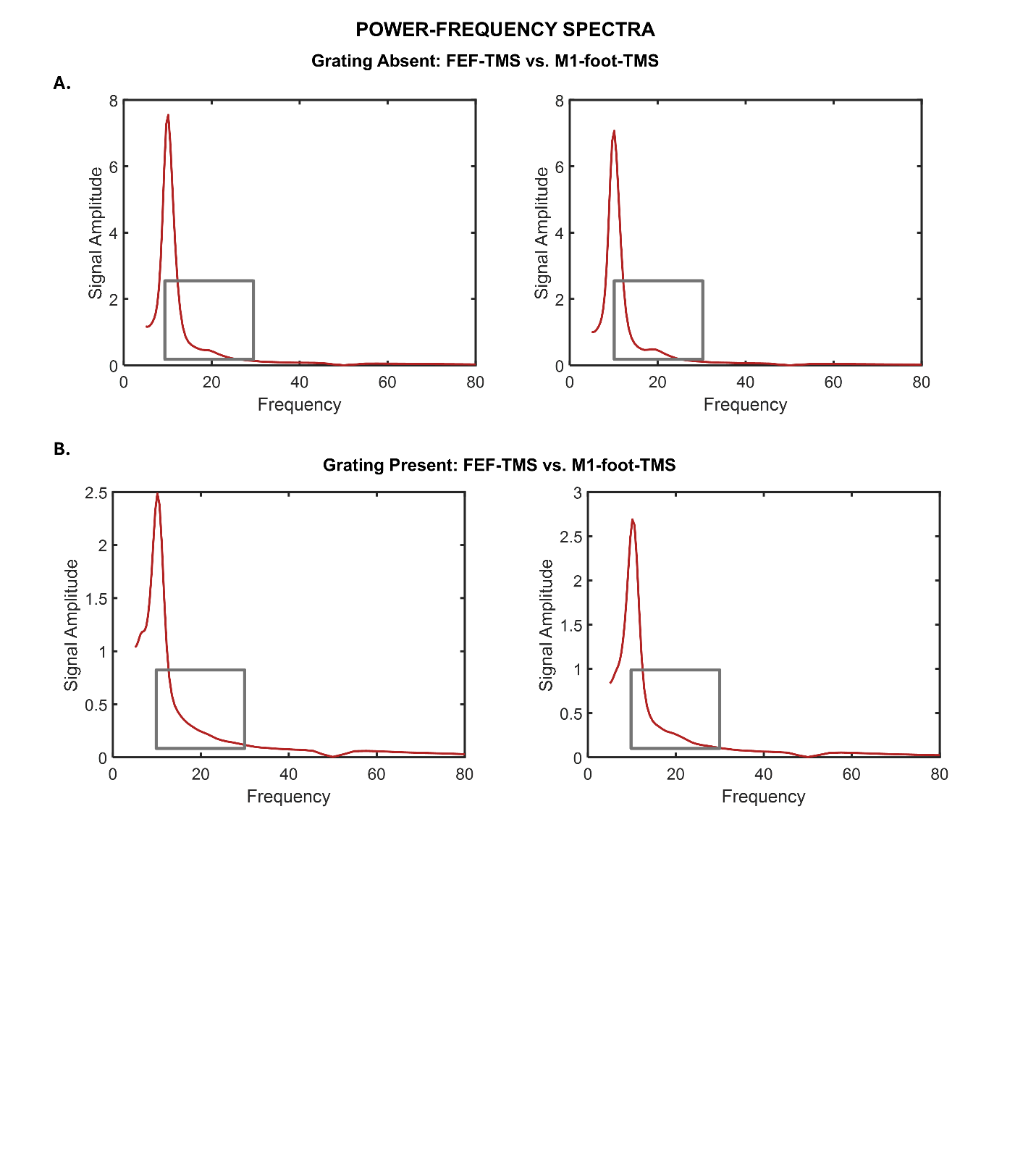 |
| --- |

**S3 Fig.** **Power-frequency spectra** showing the presence of high alpha-low beta band activity which Is a prerequisite for phase-analyses in these frequency bands. **A.** Grating absent condition. Power-frequency spectrum for the FEF-TMS (left) and M1-TMS condition (right) averaged across participants. Grey square represents the frequency window of interest. **B.** Grating present condition. Power-frequency spectrum for the FEF-TMS (left) and M1-TMS condition (right) averaged across participants. Grey square represents the frequency window of interest.
