# Supplementary material for: Top-down and bottom-up interactions rely on nested brain oscillations to shape rhythmic visual attention sampling
Source: PLoS Biol. 2025 Apr 10;23(4):e3002688. doi: 10.1371/journal.pbio.3002688 (PMC12037075; doi:10.1371/journal.pbio.3002688)
Supplement: S4 Fig — No evidence for between-block differences in power or ITPC (only no-TMS trials included) ruling out potential confounding effects of block on the TMS results. (A) Left panel: Raw differences in time-frequency plots of the posterior electrodes (O2, O1, POz, Oz, PO8, PO7, PO4, PO3) between different blocks of the grating-absent condition (GRATING−). Frequency range for the analysis (y-axis) is from 5–80 Hz. Time range for the analysis (x-axis) is from −200–1,000 ms, where 0 is the time point of the grating onset. Right panel: Z-scores of the permutation-based analysis between different block of the grating-absent condition. No significant clusters were identified. (B) Left panel: Raw differences in time-frequency plots of the posterior electrodes (O2, O1, POz, Oz, PO8, PO7, PO4, PO3) between different blocks of the grating-present condition (GRATING+). Frequency range for the analysis (y-axis) is from 5–80 Hz. Time range for the analysis (x-axis) is from −200–1,000 ms, where 0 is the time point of the grating onset. Right panel: Z-scores of the permutation-based analysis between different blocks of the grating-present condition. No significant clusters were identified. (C) Left panel: Inter-trial phase coherence (ITPC) differences of the posterior cluster in the stimulated (right) hemisphere (electrodes: O2, POz, Oz, PO8, PO4) between different blocks of the grating-absent condition (GRATING−). The time range for the analysis (x-axis) is from −200–1,000 ms, where 0 point is the timing of the TMS pulse. The frequency (y-axis) is from 5–80 Hz. Right panel: Z-scores of the permutation-based statistical analysis between different blocks of the grating-absent condition (GRATING−). No significant clusters were identified. (D) Left panel: ITPC differences of the posterior cluster in the stimulated (right) hemisphere (electrodes: O2, POz, Oz, PO8, PO4) between different blocks of the grating-present condition (GRATING+). The time range for the analysis (x-axis) is from −200–1 [file pbio.3002688.s004.docx]

| 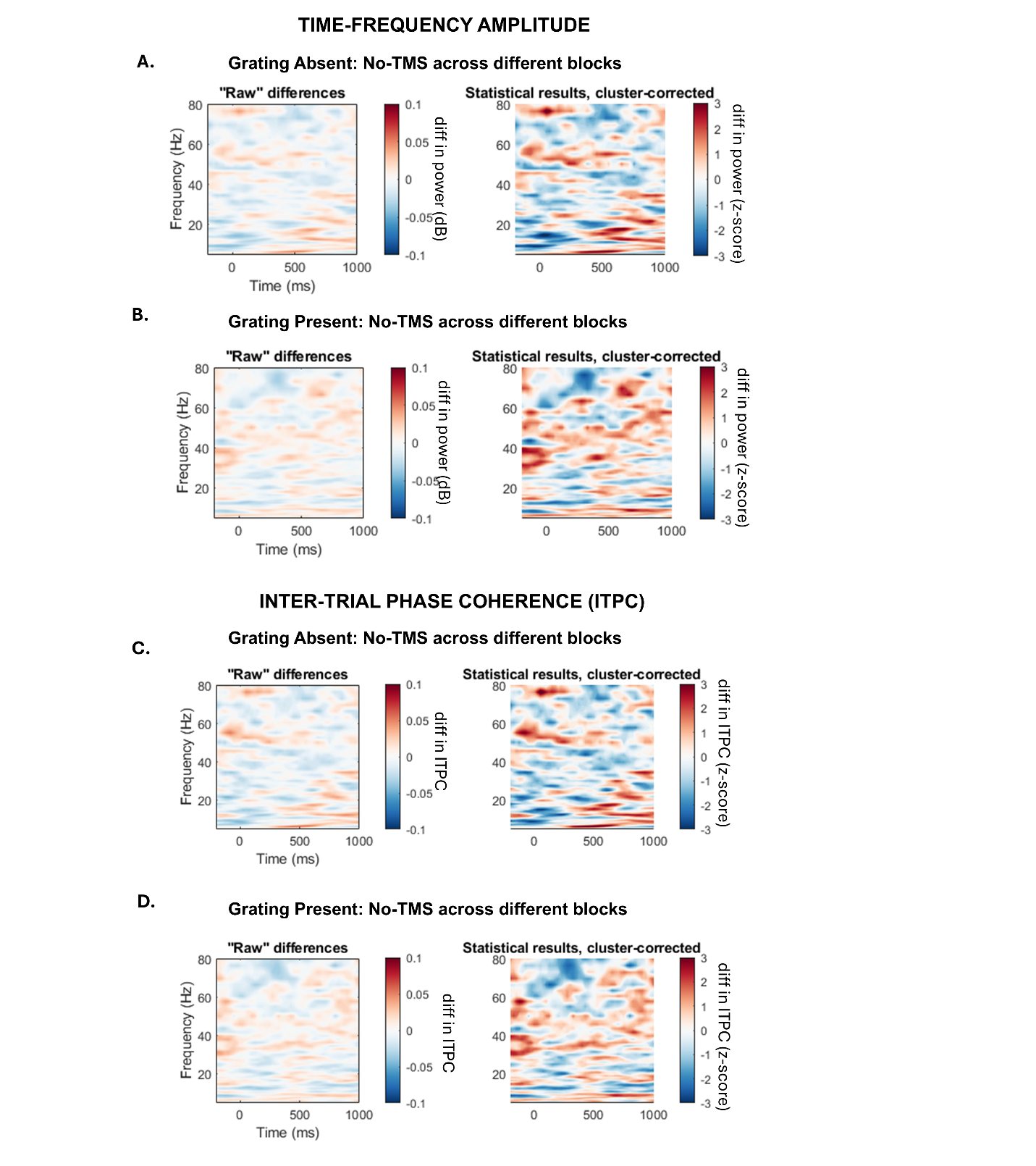 |
| --- |

**S4 Fig.** **Time-Frequency power and ITPC Analysis for the no-TMS trials across different blocks: Control analysis**. No evidence for between-block differences in power or ITPC (only no TMS trials included) ruling out potential confounding effects of block on the TMS results. **A.** Left panel: Raw differences in time-frequency plots of the posterior electrodes (O2, O1, POz, Oz, PO8, PO7, PO4, PO3) between different blocks of the grating absent condition (GRATING-). Frequency range for the analysis (y-axis) is from 5-80Hz. Time range for the analysis (x-axis) is from -200 to 1000 ms, where 0 is the time point of the grating onset. Right panel: Z-scores of the permutation-based analysis between different block of the grating absent condition. No significant clusters were identified. **B.** Left panel: Raw differences in time-frequency plots of the posterior electrodes (O2, O1, POz, Oz, PO8, PO7, PO4, PO3) between different blocks of the grating present condition (GRATING+). Frequency range for the analysis (y-axis) is from 5-80Hz. Time range for the analysis (x-axis) is from -200 to 1000 ms, where 0 is the time point of the grating onset. Right panel: Z-scores of the permutation-based analysis between different blocks of the grating present condition. No significant clusters were identified. **C.** Left panel: Inter-trial phase coherence (ITPC) differences of the posterior cluster in the stimulated (right) hemisphere (electrodes: O2, POz, Oz, PO8, PO4) between different blocks of the grating absent condition (GRATING-). The time range for the analysis (x-axis) is from -200 to 1000 ms, where 0 point is the timing of the TMS-pulse.The frequency (y-axis) is from 5-80 Hz. Right panel: Z-scores of the permutation-based statistical analysis between different blocks of the grating absent condition (GRATING-). No significant clusters were identified. **D.** Left panel: Inter-trial phase coherence (ITPC) differences of the posterior cluster in the stimulated (right) hemisphere (electrodes: O2, POz, Oz, PO8, PO4) between different blocks of the grating present condition (GRATING+). The time range for the analysis (x-axis) is from -200 to 1000 ms, where 0 point is the timing of the TMS-pulse.The frequency (y-axis) is from 5-80 Hz. Right panel: Z-scores of the permutation-based statistical analysis between different blocks of the grating present condition (GRATING+). No significant clusters were identified. diff = difference; dB = decibel; Hz=hertz; t=time.
