# Supplementary material for: Top-down and bottom-up interactions rely on nested brain oscillations to shape rhythmic visual attention sampling
Source: PLoS Biol. 2025 Apr 10;23(4):e3002688. doi: 10.1371/journal.pbio.3002688 (PMC12037075; doi:10.1371/journal.pbio.3002688)
Supplement: S5 Fig — (A) Left: Grating-absent Condition. Connectivity matrix measured by the wPL) across all electrodes included in the regions of interest for the grating-absent condition in the higher alpha/lower beta range (13–20 Hz), in the right (stimulated) hemisphere. No significant differences in interregional coupling across different experimental blocks were identified. Right: Grating-present Condition. Connectivity matrix measured by the wPLI across all electrodes included in the regions of interest for the grating-present condition in the higher alpha/lower beta range (13–20 Hz), in the right (stimulated) hemisphere. Red/blue ink indicates significant differences in interregional coupling across different experimental blocks. (B) Grating-absent condition. Left panel: Raw differences in modulation index (MI) plots of the posterior cluster in the stimulated (right) hemisphere (electrodes: O2, POz, Oz, PO8, PO4) between different block of the no-TMS trials. The frequency for lower phase frequency is shown in the y-axis. The frequency for the higher gamma amplitude- in the x-axis. Right panel: Z-scores of the permutation-based analysis between different experimental blocks. No significant clusters were identified. (C) Grating-present condition. Left panel: Raw differences in MI plots of the posterior cluster in the stimulated (right) hemisphere (electrodes: O2, POz, Oz, PO8, PO4) between different block of the no-TMS trials. The frequency for lower phase frequency is shown in the y-axis. The frequency for the higher gamma amplitude in the x-axis. Right panel: Z-scores of the permutation-based analysis between different experimental blocks. No significant clusters were identified. (DOCX) [file pbio.3002688.s005.docx]

| 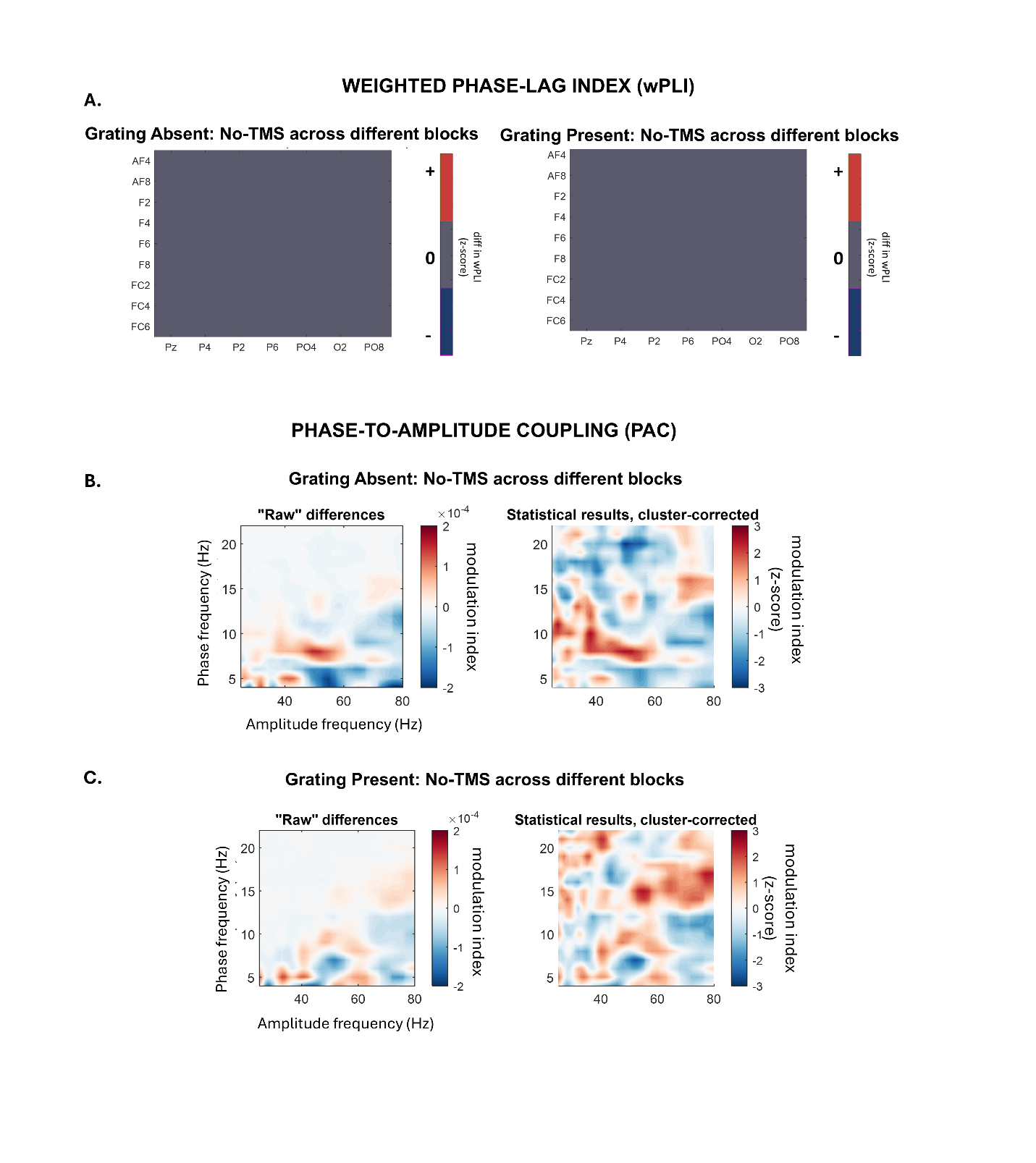 |
| --- |

***S5 Fig. Weighter phase-lag index (Wpli) and Phase-to-amplitude coupling (PAC) for the no-TMS trials across different blocks: Control analysis.*** No evidence for between-block differences in wPLI or PAC (only no TMS trials included) ruling out potential confounding effects of block on the TMS results*.* **A**. Left: Grating absent Condition. Connectivity matrix measured by the weighted phase lag index (wPLI) across all electrodes included in the regions of interest for the grating absent condition in the higher alpha/lower beta range (13-20 Hz), in the right (stimulated) hemisphere. No significant differences in interregional coupling across different experimental blocks were identified. Right: Grating present Condition. Connectivity matrix measured by the weighted phase lag index (wPLI) across all electrodes included in the regions of interest for the grating present condition in the higher alpha/lower beta range (13-20 Hz), , in the right (stimulated) hemisphere. Red/blue ink indicates significant differences in interregional coupling across different experimental blocks. **B. Grating Absent condition.** Left panel: Raw differences in modulation index (MI) plots of the posterior cluster in the stimulated (right) hemisphere (electrodes: O2, POz, Oz, PO8, PO4) between different block of the no-TMS trials. The frequency for lower phase frequency is shown in the y-axis . The frequency for the higher gamma amplitude- in the x-axis. Right panel: Z-scores of the permutation-based analysis between different experimental blocks. No significant clusters were identified. **C. Grating Present condition.** Left panel: Raw differences in modulation index (MI) plots of the posterior cluster in the stimulated (right) hemisphere (electrodes: O2, POz, Oz, PO8, PO4) between different block of the no-TMS trials. The frequency for lower phase frequency is shown in the y-axis . The frequency for the higher gamma amplitude in the x-axis. Right panel: Z-scores of the permutation-based analysis between different experimental blocks. No significant clusters were identified.
