# Supplementary material for: Top-down and bottom-up interactions rely on nested brain oscillations to shape rhythmic visual attention sampling
Source: PLoS Biol. 2025 Apr 10;23(4):e3002688. doi: 10.1371/journal.pbio.3002688 (PMC12037075; doi:10.1371/journal.pbio.3002688)
Supplement: Attachment — Submitted filename: Response to reviewers R1.docx [file pbio.3002688.s008.docx]

------------------------------------
REVIEWS:

Reviewer #1: The article entitled "Top-down and bottom-up interactions rely on nested brain oscillations" by Trajkovic et al. provides supporting evidence for frequency-specific top-down modulation of bottom-up sensory signals in human participants.
Using single pulse transcranial magnetic stimulation (TMS) of the Frontal Eye Field (FEF), they show increased phase realignment of activity at alpha/beta frequency for ipsilateral occipito-parietal sensors and increased alpha/beta phase-synchronization between frontal and occipito-parietal sensors. These effects are significant with or without visual stimulation but phase-amplitude coupling between frontal and occipital oscillatory activity is only significant during visual stimulation and shows that occipital gamma frequency power is aligned to alpha/beta phase.
This article seems at first like an implementation of their previous study with Joachim Gross (Veneiro et al., NatCommun2022) reporting that TMS activation of FEF induces beta phase-reset over occipital sites, although it brings important information that could not be analyzed from the 2022 study.
The advance resides in the fact that they causally tested previous non-human primate observations of cross-frequency coupling between top-down beta and bottom-up gamma frequency oscillations (e.g. Richter et al., 2017).
They used TMS in the presence versus absence of sensory activity and explore frequency interactions up to 80Hz thus allowing them to look for effect of top-down alpha/beta on bottom-up gamma. However, the functional connectivity measure used (wPLI) is not directional.

The paper reads well and results are clearly reported, although some aspects of the results shall be discussed further.
In addition, results on TMS effects on behavioral performance shall also be reported and discussed.

The points to address are detailed below:
**1.**      PAC of alpha/beta to gamma oscillations is associated to a mechanism through which top-down/feedback modulates bottom-up/feedforward signals for optimal performance. However, results on behavioral performance are not presented at all. For the interest of the reader, I strongly recommend the authors to report the behavioral effect of TMS when grating present (either on performance or on reaction time by contrast with M1foot-TMS and/or without TMS) and include discussion about these and comparison with literature. As FEF-TMS is reported to enhance alpha/beta phase synchronization and occipital gamma power, one clearly expect behavior to follow e.g. Rohenkohl et al. Neuron 2018; Parto-Dezfouli et al., Cell Rep. 2023.

We agree with this reviewer (and reviewer 3, see below) that the functional role of the top-down signal is indeed to mold the bottom-up signaling for modulating sensory processing with impact on behavioural performance. We have already revealed these behavioural effects in our previous study ^1^, where we established that FEF-TMS is changing perceptual accuracy, which required testing many different time-points (via dense behavioral sampling) between the TMS pulse and the target stimulus (revealing perceptual performance to oscillate at the phase-reset beta oscillations). Having established these effects on behavioral performance previously, we here attempted to optimize our design to collect as many clean (target-free) EEG-trials as possible within a reasonable timeframe for the participants and to estimate the top-down and bottom-up signals in EEG with a good S/N ratio. That is, due to the already long duration of our experiment, we decided against testing different timings between the TMS pulse and the target stimulus (i.e., glitch in the movement of the grating) and opted for the TMS pulse to coincide with the presentation of the target stimulus. Opting for this single time point, the task itself served the purpose of keeping the participants engaged throughout the experiment. However, the simultaneity of FEF-activation and target presentation prevents TMS-induced activation to interact with target processing.

Hence, our current experimental design was optimized for tracking EEG-changes in response to FEF-activation and sensory input but not the associated behavioral effects. Importantly, because of i) the close match of the present with the previous design of Veniero et al, ^1^, and ii) the fact that we here replicate their FEF-TMS induced phase-realignment at high alpha/low beta frequency over occipital EEG-sites ^1^, while iii) showing this to interact with visual gamma-activity, we would expect behavioral performance to co-fluctuate with these effects, i.e. not to differ from ^1^. We now made this clearer in the manuscript.

2.      On Figure 4B and C, it is clear that FEF-TMS effects on alpha/beta phase-synchronization between frontal and parieto-occipital sensors differ. In Grating+ condition, only AF4-8 show increased synchronization with almost all parieto-occipital sensors, while in Grating- condition, more than half of frontal sensors show increased synchronization with PO4-8-2 only. These topographic differences shall be discussed further.

We thank the reviewer for this important point. It is widely accepted that the effects of the TMS depend on the prior state of the brain, with brain state-dependent influences on the propagation of TMS-induced local action potentials ^2–5^. Accordingly, it is conceivable that network activation patterns differ between states where sensory input is differentially manipulated (Grating+ versus Grating – condition)^6,7^. Consequently, the spread of the TMS pulse can depend on prior network activation, which could explain why the patterns of connectivity differences vary between the stimulus present versus absent condition (see also ^8^). We now included this as an additional notable point in the manuscript.

3.      The authors refer to prominent theories of oscillatory communication (paragraph lines282-295 p10). It would make the discussion on nested oscillations more appealing to the reader (and potentially increase the impact of the article) when considering more alternative (although not incompatible) views e.g. Vinck et al., Neuron 2023; Bastos et al., PNAS 2020.

We thank the reviewer for these interesting suggestions. Our results are indeed compatible with these alternative accounts pointed out by the reviewer.

This includes the predictive routing account by Bastos et al. ^9^, where feedback signaling carries expectations about sensory stimuli through alpha/beta rhythms, while feedforward signaling through gamma activity becomes amplified during unpredicted and surprising inputs.

The new framework of Vinck et al ^10^ does also seem compatible with our findings, for two reasons. First, while it challenges the communication-through-coherence hypothesis and proposes that coherence is a consequence of communication via other mechanisms such as linear signal transfer and hence that coherence does not reflect communication and information processing per se, it does accommodate phase-shifts as a means for communication. More specifically, the framework proposes that even though coherence is not a physiologically plausible mechanism for mediating communication, the phase shifts could reliably achieve selective communication at relatively short timescales. This is in line with our results on phase alignment. Second, the framework suggests that neural communication might rely on resonance such that communication is facilitated when a sending population shifts its energy toward the receiver’s population intrinsic resonance peak. This also would seem compatible with our findings, as resonance-driven rhythms engaged in feedback processing involving different timescales and various oscillatory mechanisms require non-linear integration processes, such as cross-frequency coupling. Finally, this latter point raises an intriguing alternative that should be considered, namely that the causal evidence for nesting of the bottom-up gamma activity in the alpha/beta-band cycles might reflect the intrinsic resonance properties of the sender’s (PFC gamma) influence on the receiver’s (posterior alpha, beta) properties.

We have now included these points in our revised manuscript (please see discussion).

Reviewer #2: This is a very strong study, addressing an important question in cognitive neuroscience with very robust methods. I only have couple of very minor points:

1. Sample size - 30 participants were tested. What was this based on? Were power calculations carried out prior to the study.

We thank the reviewer for this question, as it is indeed important to justify our sample size. We did run an a-priori power calculation to estimate the sample size, based on our within-participants design (STIMULATION SITE: FEF vs. M1_foot;_ TMS pulse present (TMS+) vs. absent (TMS-)), using a medium effect size and 0.90% power, which yielded a sample size of n=30 participants. In addition, we did run a post-hoc analysis to confirm that our sample size is adequate for the used analysis approach, based on power analysis using data simulation for cluster-based permutation tests. With this analysis, we confirmed that our sample is adequate, where a minimum sample size of 22 participants was required to obtain 90% power to detect differences in time-frequency data between two conditions for a within-subject design. We now specified this in the manuscript.

2. TMS intensity - could this be expanded upon? Is there any reason to expect that low vs high intensities would have different effects, as has been shown with single pulse TMS?

As described in the manuscript, we opted for a fixed intensity (65% of the MSO) since it was this TMS-intensity that revealed effects of FEF-stimulation on visual processes not only in our previous study ^1^, but also other studies on FEF-stimulation by TMS ^11,12^. Taking into account research exploring dose-dependent network effects of FEF-TMS (e.g, ^8^), it is conceivable that we would have found effects of FEF-TMS on visual activity with lower intensities too, given that Ruff et al.^8^ reported that TMS-intensities at 40-55% of MSO over FEF can also spread to visual areas or even outweigh effects at higher intensities, depending on the visual area under investigation (calcarine vs occipital pole) and presence or absence of visual input. This has been added to the manuscript, alongside a comment that it would be of interest to investigate dose-dependency of FEF-TMS on oscillatory activity in future studies.

Reviewer #3: In "Top-down and bottom-up interactions rely on nested brain oscillations", Trajkovic and colleagues build on considerable human and NHP work demonstrating that top-down influences arise via alpha/beta synchrony while bottom-up information is carried via gamma synchrony and ask how these two signals are related. They specifically examine whether these two signals are coordinated via increased coupling between alpha/beta phase and gamma amplitude. The results are relatively straightforward and make a theoretical advance into our understanding of top-down influences on perception. I have a few concerns that the authors should address, which I detail below.

We thank the reviewer for all their important suggestions regarding control analyses, sanity checks and clarifications to rule out caveats that are associated with phase-based analyses as used in the present work. We have run these analyses/ sanity checks, which support our conclusions, and report them in the manuscript or in the Supporting information for convenience of the reader, see text and Figures S2-S5

(1) One potential concern about comparing phase-based connectivity measures (here, wPLI) that calculate the magnitude of the phase lag across conditions is that any differences between conditions could be due to a difference in the preferred phase angle difference between electrode sites, which is not necessarily indicative of increased connectivity. For more details, please refer to chapter 26 (~page 350 in the hardcover edition) of ref [65]. It would be prudent for the authors to rule out this possibility by checking the phase lag angle difference in both conditions and ensuring that they are not significantly different.

We agree that it is important to verify that potential differences in the preferred phase lag angle differences between the electrode sites are not responsible for the obtained connectivity differences. We now looked at potential differences between the electrodes and frequencies where we found significant connectivity changes and included this control analysis in Supplementary Materials (See Figure S2). In sum, we did not find any significant modulation of the preferred phase-angle differences between electrode sites across different conditions, thus ruling out the possibility that this might guide our effects.

(2) There are a few additional important details about the methods and analyses that are missing. First, the time windows and frequency ranges reported vary across different analyses with little to no justification. The effects of TMS on alpha/beta band ITPC lasted for 250 or 400ms, depending on condition, but connectivity was calculated over a 300ms post-TMS window wPLI ….:

We are sorry for the confusion and for not making this point explicit. We opted for 300 ms, because it’s the timing where the effect should still be present in both conditions, while at the same time having at least 4 entire cycles of the high alpha/low beta activity to assess the connectivity. We now explain this in the manuscript.

…., and PAC was tested in a 250ms post-TMS window in a wider frequency range than was reported earlier in the manuscript. There is also an incongruity between the gamma range in which PAC was tested (30-80Hz) and the gamma range in which significant results were observed (60-90Hz).

PAC was tested in this time-window because it is the same window where we found the ITPC effect in the GRATING+ condition (where we also expected a higher PAC). As per frequency differences, gamma range was indeed defined as 30-80 Hz (as per other analysis), while during the subsequent analysis steps the data for the amplitude-coupling (high) frequencies gets filtered in steps of 10Hz (with the final frequency window being from 80-90Hz). This is why we ended up with the gamma range up to 90 Hz.

However, we do agree that having a different time window/frequency range for the connectivity versus PAC analysis might not be optimal. Therefore, we have now repeated the analysis for the 300 ms time window and 30-80 Hz frequency range, which yielded the same results. We now include this new analysis in the manuscript (see Figure 5).

Second, a substantial number of trials are from the no-TMS condition, which occurred in both the FEF-TMS and M1-TMS blocks. I could not find any results from these trials. It would be instructive to confirm that the gamma power, alpha/beta ITPC, and alpha/beta-gamma PAC results did not differ between the no-TMS trials in the two blocks to
rule out a general effect of the blocks that is not specific to the TMS pulse.

We agree with the reviewer that it is important to affirm that there aren’t any between-blocks differences that might influence our effects. This is why we now compared no-TMS trials from different blocks, as suggested, and included these control analyses in the Supplementary Materials (see Figure S4 and Figure S5). In summary, we did not find any significant between-block differences in gamma power, alpha/beta ITPC, wPLI and alpha/beta-gamma coupling. Hence, this analysis further strengthens our initial conclusion of differences being attributable to FEF-activation unconfounded by a general effect of blocks.

Third, the authors should provide a justification for the brief time window used to calculate PAC, as, depending on the method, some authors advise using a window of up to 10s.

As evidenced by the ITPC differences, and prior research on the duration of the sp-TMS effects, we cannot expect the effect to be longer than the effect on ITPC, which was around 300 ms. However, we acknowledge that this time window for the PAC analysis might be considered short. Hence, we also calculated an alternative measure of PAC to confirm the validity of our initial findings. Specifically, we calculated generalized eigendecomposition-based cross-frequency coupling. Briefly, this method uses eigendecompositions to extract a component that maximizes the difference between high-frequency activity that appears during peaks of a low-frequency oscillation (in our case, we chose the frequencies identified as significant in out prior PAC analysis: 13-20 Hz), versus high-frequency activity that is unrelated to the low-frequency oscillation. This analysis confirmed our previous results, by demonstrating that, in the GRATING+ condition, there was indeed a significant increase in the gamma activity locked to the low frequency peaks in the high gamma range, lasting for ~300 ms (see Figure below). The high convergence of results between the two independent analysis approaches supports our initial result to represent a true effect rather than a consequence of using an inadequate time window for this analysis.

(3) There are a few caveats to the ITPC analysis that the authors need to consider. First, phase measures are only valid when power is sufficient. It would be a good sanity check to see the power at the frequencies of interest, perhaps as a supplementary figure.

We thank the reviewer for this suggestion, and we now include power-frequency spectra for each condition for sanity checks (see Figure S3).

Second, related to point (2) above, it is not clear how many trials are in each of the conditions of interest. ITPC is sensitive to unbalanced trial numbers, so the authors should ensure that the number of trials is comparable between M1 and FEF conditions and report this information.

We thank the reviewer for this suggestion. We can confirm that the trial number across different stimulation sites (FEF vs. control site) was not statistically different for both the Grating present (Mfef = 69.6 SE=1.54; Mctrl = 71.4 SE = 0.86 , t= 1.228 , p = .229) and Grating absent condition (Mfef = 62.8 SE=1.72; Mctrl = 62.9 SE = 1.68, t= 0.057 , p = .955).

This information is now included in the revisions.

(4) Presumably the pattern of results described here is exciting because ultimately it is adaptive for behavior; yet, there is no discussion of how TMS, which was intended to emulate top-down signals, affected behavior. The authors have a behavioral measure that might be useful in glitch detection performance. They even carefully titrate behavior prior to the experiment to ensure that participants correctly detect the glitch 70% of the time. Wouldn't one expect increased performance in the FEF TMS condition? How should our interpretation of the results change if this is not the case?

We agree with this reviewer (and reviewer 1, see above) that the functional role of the top-down signal is indeed to mold the bottom-up signaling for modulating sensory processing with impact on behavioral performance. We have already revealed these behavioural effects in our previous study ^1^, where we established that FEF-TMS is changing perceptual accuracy, which required testing many different time-points (via dense behavioral sampling) between the TMS pulse and the target stimulus (revealing perceptual performance to oscillate at the phase-reset beta oscillations). Having established these effects on behavioral performance previously, we here attempted to optimize our design to collect as many clean (target-free) EEG-trials as possible within a reasonable timeframe for the participants to estimate the top-down and bottom-up signals in EEG with a good S/N ratio. That is, due to the already long duration of our experiment, we decided against testing different timings between the TMS pulse and the target stimulus (i.e., glitch in the movement of the grating) and opted for the TMS pulse to coincide with the presentation of the target stimulus. Opting for this single time point, the task itself served the purpose of keeping the participants engaged throughout the experiment. However, the simultaneity of FEF-activation and target presentation prevents TMS-induced activation to interact with target processing.

Hence, our current experimental design was optimized for tracking EEG-changes in response to FEF-activation and sensory input but not the associated behavioral effects. Importantly, because of i) the close match of the present with the previous design of Veniero et al, ^1^, and ii) the fact that we here replicate their FEF-TMS induced phase-realignment at high alpha/low beta frequency over occipital EEG-sites (1), while iii) showing this to interact with visual gamma-activity, we would expect behavioral performance to co-fluctuate with these effects, i.e. not to differ from ^1^. We now made this clearer in the manuscript.

(5) Some minor points:
*       There is some relevant work by Riddle and colleagues (Riddle et al., 2019, JoCN; Riddle et al., 2020, Current Biology) that the authors should consider including in their introduction/discussion. We thank the reviewer for pointing out these indeed relevant studies. We now included them in our manuscript.
*       The sentence starting on p. 6, line 181 is confusing and potentially misleading; one can take it to mean that gamma-band activity over posterior sites is related to phase realignment. We are sorry for the confusion, and we now rephrased this sentence.
*       Figure 1 - I believe "ANS" should be "AND" We now corrected this typo.
*       The citation format in parts of the Methods section is inconsistent with the rest of the manuscript. We now corrected the citation format.

**References**

1.	Veniero D, Gross J, Morand S, Duecker F, Sack AT, Thut G. Top-down control of visual cortex by the frontal eye fields through oscillatory realignment. *Nat Commun*. 2021;12(1):1757. doi:10.1038/s41467-021-21979-7

2.	Silvanto J, Pascual-Leone A. State-Dependency of Transcranial Magnetic Stimulation. *Brain Topogr*. 2008;21(1):1-10. doi:10.1007/s10548-008-0067-0

3.	Zokaei, Nahid M Sanjay, Husain, Masud; Feredoes, Eva. Causal Evidence for a Privileged Working Memory State in Early Visual Cortex | Journal of Neuroscience. Accessed December 11, 2024. https://www.jneurosci.org/content/34/1/158

4.	Feredoes E, Heinen K, Weiskopf N, Ruff C, Driver J. Causal evidence for frontal involvement in memory target maintenance by posterior brain areas during distracter interference of visual working memory. *Proc Natl Acad Sci U S A*. 2011;108(42):17510-17515. doi:10.1073/pnas.1106439108

5.	Sack AT, Paneva J, Küthe T, et al. Target Engagement and Brain State Dependence of Transcranial Magnetic Stimulation: Implications for Clinical Practice. *Biol Psychiatry*. 2024;95(6):536-544. doi:10.1016/j.biopsych.2023.09.011

6.	McCarthy G, Spicer M, Adrignolo A, Luby M, Gore J, Allison T. Brain activation associated with visual motion studied by functional magnetic resonance imaging in humans. *Human Brain Mapping*. 1994;2(4):234-243. doi:10.1002/hbm.460020405

7.	Kanwisher N, Wojciulik E. Visual attention: Insights from brain imaging. *Nat Rev Neurosci*. 2000;1(2):91-100. doi:10.1038/35039043

8.	Ruff CC, Blankenburg F, Bjoertomt O, et al. Concurrent TMS-fMRI and Psychophysics Reveal Frontal Influences on Human Retinotopic Visual Cortex. *Current Biology*. 2006;16(15):1479-1488. doi:10.1016/j.cub.2006.06.057

9.	Bastos AM, Lundqvist M, Waite AS, Kopell N, Miller EK. Layer and rhythm specificity for predictive routing. *Proceedings of the National Academy of Sciences*. 2020;117(49):31459-31469. doi:10.1073/pnas.2014868117

10.	Vinck M, Uran C, Spyropoulos G, et al. Principles of large-scale neural interactions. *Neuron*. 2023;111(7):987-1002. doi:10.1016/j.neuron.2023.03.015

11.	Grosbras MH, Paus T. Transcranial magnetic stimulation of the human frontal eye field facilitates visual awareness. *Eur J Neurosci*. 2003;18(11):3121-3126. doi:10.1111/j.1460-9568.2003.03055.x

12.	O’Shea J, Muggleton NG, Cowey A, Walsh V. Timing of target discrimination in human frontal eye fields. *J Cogn Neurosci*. 2004;16(6):1060-1067. doi:10.1162/0898929041502634
